# Myeloid antigens in childhood lymphoblastic leukemia:clinical data  point to regulation of CD66c distinct from other myeloid antigens

**DOI:** 10.1186/1471-2407-5-38

**Published:** 2005-04-12

**Authors:** Tomas Kalina, Martina Vaskova, Ester Mejstrikova, Jozef Madzo, Jan Trka, Jan Stary, Ondrej Hrusak

**Affiliations:** 1 Department of Immunology, Charles University 2nd Medical School, Prague, Czech Republic; 2 Department of Pediatric Hematology and Oncology, Charles University 2nd Medical School, Prague, Czech Republic; 3 CLIP – Childhood Leukemia Investigation Prague Czech Republic

## Abstract

**Background:**

Aberrant expression of myeloid antigens (MyAgs) on acute lymphoblastic leukemia (ALL) cells is a well-documented phenomenon, although its regulating mechanisms are unclear. MyAgs in ALL are interpreted e.g. as hallmarks of early differentiation stage and/or lineage indecisiveness. Granulocytic marker CD66c – Carcinoembryonic antigen-related cell adhesion molecule 6 (CEACAM6) is aberrantly expressed on ALL with strong correlation to genotype (negative in TEL/AML1 and MLL/AF4, positive in BCR/ABL and hyperdiploid cases).

**Methods:**

In a cohort of 365 consecutively diagnosed Czech B-precursor ALL patients, we analyze distribution of MyAg+ cases and mutual relationship among CD13, CD15, CD33, CD65 and CD66c. The most frequent MyAg (CD66c) is studied further regarding its stability from diagnosis to relapse, prognostic significance and regulation of surface expression. For the latter, flow cytometry, Western blot and quantitative RT-PCR on sorted cells is used.

**Results:**

We show CD66c is expressed in 43% patients, which is more frequent than other MyAgs studied. In addition, CD66c expression negatively correlates with CD13 (p < 0.0001), CD33 (p = 0.002) and/or CD65 (p = 0.029). Our data show that different myeloid antigens often differ in biological importance, which may be obscured by combining them into "MyAg positive ALL". We show that unlike other MyAgs, CD66c expression is not shifted from the onset of ALL to relapse (n = 39, time to relapse 0.3–5.3 years). Although opposite has previously been suggested, we show that CEACAM6 transcription is invariably followed by surface expression (by quantitative RT-PCR on sorted cells) and that malignant cells containing CD66c in cytoplasm without surface expression are not found by flow cytometry nor by Western blot in vivo. We report no prognostic significance of CD66c, globally or separately in genotype subsets of B-precursor ALL, nor an association with known risk factors (n = 254).

**Conclusion:**

In contrast to general notion we show that different MyAgs in lymphoblastic leukemia represent different biological circumstances. We chose the most frequent and tightly genotype-associated MyAg CD66c to show its stabile expression in patients from diagnosis to relapse, which differs from what is known on the other MyAgs. Surface expression of CD66c is regulated at the gene transcription level, in contrast to previous reports.

## Background

Although expression of surface markers in acute lymphoblastic leukemia (ALL) parallels that of normal hematopoietic precursors, several markers of myeloid lineage are found on ALL lymphoblasts. This phenomenon is referred to as "aberrant expression". The issue of the regulatory mechanisms that allow it has been addressed repeatedly throughout the recent 40 years [1,2]. Although several hypotheses stressing either possible lineage indecisiveness or genetic misprogramming have been raised, the phenomenon is still not fully understood. We and others have shown that the myeloid antigen CD66c is very frequently aberrantly expressed in B-precursor ALL, however, a large study showing its frequency in the light of other myeloid antigens has been missing. CD66c expression was found on cases of childhood and adult ALL in strong correlation with nonrandom genetic changes (BCR/ABL positivity [3], hyperdiploidy and TEL/AML1 negativity [4], reviewed in [5]).

CD66c (CEACAM6, previously called Nonspecific cross-reacting antigen, NCA 90/50 and KOR-SA3544 antigen) is a member of the carcinoembryonic antigen family. This heavily glycosylated molecule consists of two constant Ig-like domains and one variable Ig-like domain and it is anchored to the membrane via its glycosylphosphatidylinositol (GPI). Within the hematopoietic system, CD66c expression is limited to granulocytes and its precursors [3,6], where it serves homotypic and heterotypic adhesion [7], Ca2+ mediated signaling [8] and is markedly upregulated from intracellular stores after activation [9].

It is also found in epithelia of various organs [7]. Upregulation of CD66c is an early molecular event in transformation leading to colorectal tumors [10]. It was also confirmed to inhibit anoikis (apoptotic response induced in normal cells by inadequate or inappropriate adhesion to substrate) in the in vitro model of carcinoma of colon [11] and specific silencing of this gene led to decreased metastatic potential in pancreatic adenocarcinoma [12].

Surprisingly, Sugita et al [13] reported intracellular presence of CD66c in all leukemic cell lines examined, regardless of surface presence or absence, with a different antigen distribution in cytoplasm that determined surface expression. They speculated that presence of an undisclosed transporter would target this molecule to granules and for surface expression, whereas surface CD66c^neg ^cell lines lack this transporter. This intriguing hypothesis prompted us to test whether transcription of CEACAM6 gene and/or intracellular CD66c expression is always followed by surface expression.

Uniqueness of aberrant expression of CD66c on malignant lymphoblast is exploited for diagnosis of ALL and follow-up of a minimal residual disease (MRD) using flow cytometry [14,15]. To use a marker for a MRD assessment a critical question must be addressed, whether the aberrant expression is a stable property of the malignant clone or whether it can be subject to immunophenotype shift.

In the present study we set out to address the frequency of CD66c molecule expression in childhood ALL, the regulation of CD66c expression from gene transcription to cytoplasmic and surface expression, and we follow immunophenotype stability from diagnosis to relapse. We also discuss relevance of CD66c for prognosis prediction.

## Methods

### Patients

**Figure 1:**
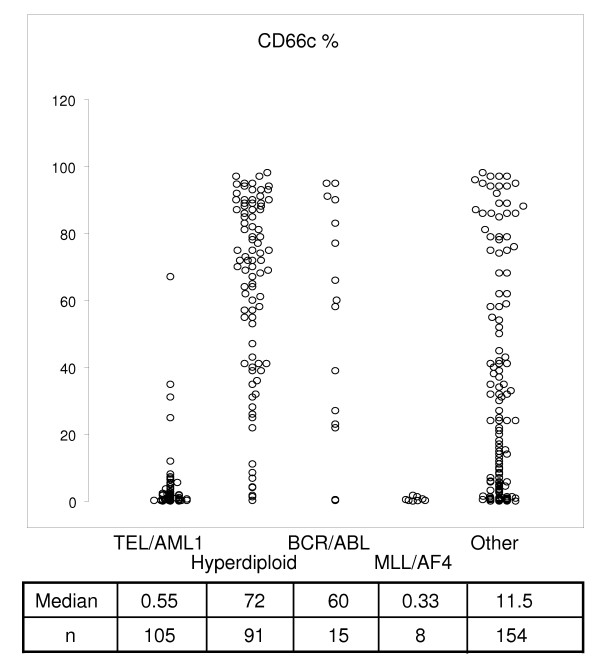
**Correlation of ALL genotype categories and percentage of CD66c positivity. **Median percentage of CD66c^pos ^blasts is listed below each genotype group. Data of consecutive unselected patients with BCP ALL (n = 373) are shown.

The cohort of all Czech children (<18 years) diagnosed with B-precursor ALL investigated in our reference laboratory from 1.5.1997 to 23.7.2004 was used for current study (n = 381). Informed consent was obtained from patients and/or their guardians. The presence of TEL/AML1, BCR/ABL and MLL/AF4 fusion genes was detected by two-round nested PCR, hyperdiploidy was assessed using DNA index flow cytometric measurement as described previously [4]. Patients' genotype and corresponding surface CD66c expression is shown in Figure 1 (genotype available in 98% of patients). For intracellular staining and FACS sorting, only samples with enough material were selected.

### Cell lines

Surface CD66c negative cell lines with typical translocation found in childhood ALL: TEL/AML1pos (REH) was kindly provided by R. Pieters (University Hospital Rotterdam), MLL/AF4pos (RS4;11) translocation and with no fusion (NALM-6) were obtained from German Cell Line collection (DSMZ, Braunschweig, Germany)

### Flow Cytometry

Flow cytometry immunophenotyping of bone marrow (BM) aspirates was performed in at diagnosis and at relapse. Routine immunophenotypic classification using panel of monoclonal antibodies (moAbs) was performed as described previously [4]. Briefly, BM samples were stained with 2-, 3- and 4-color combinations of moAbs for 15 min in darkness, erythrocytes were lysed with NH_4_Cl-containing lysing solution for 15 min, washed and data were acquired using single FACS Calibur instrument throughout the study (BD Biosciences, San Jose, CA, USA) flow cytometer. Anti-CD66c (CEACAM6) moAb used in all diagnostic and relapse measurements in this study was clone KOR-SA3544 directly labeled to FITC (Immunotech, Marseille, France). Intracellular staining was performed using Fix & Perm kit (Caltag, Burlingame, CA, USA) according to manufacturer's protocol. Acquired data was analyzed with Cell Quest (BD Biosciences) or Flow Jo (Tree Star, Ashland, OR, USA) software, lymphoblast gate was drawn based on optical scatter and CD19^pos ^blast population was selected for further analysis.

Value of 20% was chosen as a threshold of positivity as recommended by EGIL [16]. For robust prognostic significance testing, other threshold values were also tested as indicated in results.

### Cross-blocking of CD66c moAbs

Bone marrow samples of CD66c positive blasts were stained with anti-CD66c moAb clone 9A6 (Genovac, Freiburg, Germany) moAb for 15 min, erythrocytes were lysed with NH_4_Cl-containing lysing solution for 15 min, washed and sample was incubated with anti-CD66c moAb KOR-SA3544 PE moAb conjugate.

### Western blot

Samples containing 5 × 10^6 ^cells were lysed for 30 min at 4°C in 100 μl lysis buffer containing 20 mM Tris-HCl (pH 8.2), 100 mM NaCl, 50 mM NaF, 10 mM EDTA, 10 mM pyrophosphate (Na_4_P_2_O_7_) and Complete Mini EDTA-Free (protease inhibitor cocktail tablets, Roche Diagnostics, Mannheim, Germany). Debris was sedimented by centrifugation for 3 min at 13000 rpm, 0°C. Supernatants were mixed with 100 μl 2× Laemmli's SDS-polyacrylamide gel electrophoresis (PAGE) sample loading buffer, and heated for 5 min at 100°C. Proteins were fractionated by SDS-PAGE on 12.5% gels and electrophoretically transferred to PVDF membranes (Bio-Rad, Hercules, CA, USA). Membranes were blocked for 1 h in PBS (pH 7.4) containing 0.5% Tween-20 and 5% nonfat dried milk. Blots were then incubated for 1 h at room temperature with anti-KOR-SA3544 (Immunotech, Marseille, France) or anti-beta-actin (Sigma-Aldrich, Saint Louis, MO, USA) moAbs and then developed using goat anti-mouse IgG (H+L)-HRP conjugate (Bio-Rad). Immunoreactive material was then revealed by enhanced chemiluminescence (ECL, Amersham, Little Chalfont Buckinghamshire, UK) according to the manufacturer's instructions.

### Isolation of RNA and Real-Time Quantitative PCR analysis (RQ-PCR)

For RQ- PCR analysis, leukemic blasts were FACS sorted using sorting option on FACS Calibur or on FACS Aria instrument (1.1 × 10^4 ^- 4.7 × 10^5 ^cells from one patient). Isolation of RNA from FACS-sorted cells was performed using Trizol-reagent (Gibco BRL, Carlsbad, CA, USA) according to manufacturer's instructions [17]. Complementary DNA was prepared using M-MLV Reverse Transcriptase (Gibco) according to manufacturer instructions. Glycogen (Gibco) 250 μg /mL was added when initial cell number was lower than 10^5^. Quality of cDNA was verified by PCR on beta-2-microglobulin (B2M) housekeeping gene.

RQ-PCR was performed in the LightCycler™ rapid thermal cycler system (Roche Diagnostic GmbH, Mannheim, Germany), according to manufacturer's instructions, using SYBR green intercalating dye. CEACAM6 specific primers 3'-CGCCTTTGTACCAGCTGTAA and 5'-GCATGTCCCCTGGAAGGA designed by Baranov [18] were used for CEACAM6 amplification and B2M specific primers 3'-GATGCTGCTTACATGTCTCG 5'-CCAGCAGAGAATGGAAAGTC [19]were used for total cDNA quantification.

PCR amplification was carried out in 1× reaction buffer (20 mmol/L Tris-HCl, pH 8.4; 50 mmol/L KCl); and 2.0 mmol MgCl_2 _containing 200 μmol/L of each dNTP, 0.2 μmol/L of each primer, 5 μg bovine serum albumin per reaction, and 1 U of Platinum Taq DNA polymerase (all from Gibco) in a final reaction volume of 20 μL. For each PCR reaction, 2 μL of cDNA template and 2 μl of SYBR Green 5 × 10^-4 ^(FMC BioProducts, Rockland, MA, USA) fluorescent dye was included. The cycling conditions were 2.0 minutes at 95°C followed by 45 cycles of denaturation at 94°C for 5 seconds, annealing at 59°C for 30 seconds, and extension at 72°C for 15 seconds. CEACAM6 and B2M gene were amplified separately from the same cDNA, and all experiments were performed in duplicate. Melting curve analysis was performed after each run; in case of peak melting temperature shift, PCR products were verified on agarose gel electrophoresis.

### Normalized CEACAM6 Expression (CEACAM6n)

Amplification and calibration curves were generated by using affiliated software (LightCycler 3 data-analysis software; version 3.5.28; Idaho Technology Inc., Salt Lake City, UT, USA). A calibration curve for the B2M and CEACAM6 housekeeping gene was generated using the series of 10× diluted cDNA from peripheral blood granulocytes as a standard for both reactions. Crossing point (Cp) value was calculated with LightCycler 3 software using second derivative maximum method. CEACAM6n value is relative and represents a ratio of CEACAM6 to B2M (CEACAM6n = CEACAM6/ B2M). Standard cDNA from granulocytes was assigned CEACAM6n value of 1, the same aliquot of granulocytes cDNA was used throughout of study.

### Statistics

Statistical evaluation was done with Statview software, (SAS Institute Inc, NC, USA). We used Fisher's exact test, regression coefficient, Mann-Whitney test and Logrank (Mantel-Cox) test as described in text.

## Results

### Frequency of CD66c and myeloid antigen (MyAg) expression

**Figure 2:**
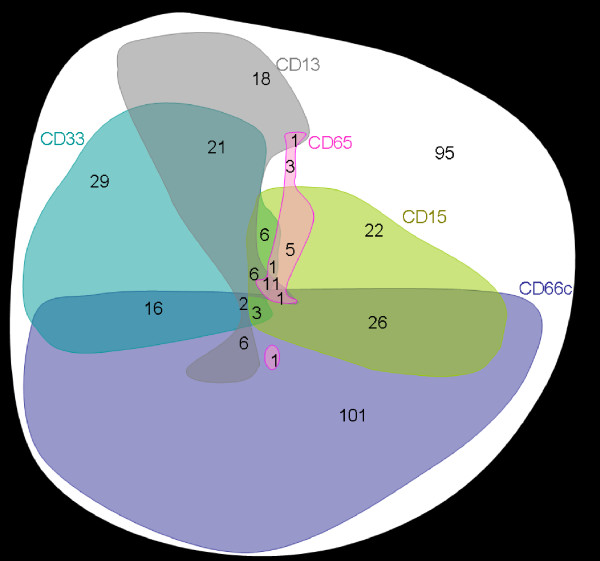
**Graphical illustration of myeloid antigen positivity in childhood B-precursor ALL. **For each antigen, positive cases are represented by a colored form. The areas of the forms roughly correspond to the frequency of positive cases (observed numbers of patients are marked in red) while the shapes are constructed to illustrate the respective coexpressions. An arbitrary cutoff value of 20% is used for all antigens. The CD66c positivity correlates with negativity of any of the following: CD33 (p = 0.002), CD13 (p < 0.0001) and CD65 (p = 0.029). There was a significant correlation between CD33 and CD13 positivity (p < 0.0001) and between CD15 and CD65 positivity (p = 0.0002) whereas the positivity of no other two antigens of the ones shown correlated significantly with each other. Total number of B-precursor cases illustrated is 365.

We selected 365 patient's samples obtained at diagnosis of B-precursor ALL with available information on the expression of MyAg CD13, CD15, CD33, CD65 and CD66c. This subcohort represents 96% of all B-precursor ALL diagnosed in the study period. The CD66c molecule was expressed on 43% cases (Table 1, cases with >20% positive blasts were considered positive). For the fraction of positive cells and correlation with genotype see [5], of note, 29% of patients expressed CD66c on more then 50% blasts. Comparison with other MyAg showed that CD66c is more frequently expressed. Coexpression of CD66c with other MyAg was not a usual finding (Table 1, Figure 2). Expression of CD13, CD33 and CD65 tended to be non-random (mutually exclusive) with CD66c (Table 1). Coexpression of CD66c with any 2 of the other MyAg was found in fewer than 4 cases in each combination. Interestingly, mutual relationship of other MyAg was random, with the exception of CD13 and CD33 coexpression (p < 0.0001) and CD15 and CD65 coexpression (p = 0.0002). The analysis was performed also at different cutoff values (10, 30 and 50 %; data not shown). The same or less significant correlations were also observed at different cutoff values.

### Cross-blocking of KOR-SA3544 clone with 9A6 clone

The moAb clone KOR-SA3544 was not included in Human Leukocyte Differentiation Antigens workshop, but was characterized by Sugita et al [13]. To prevent ambiguous interpretation of our data we extended characterization of KOR-SA3544 clone of CD66c moAb by blocking experiments on CD66c^pos ^blasts. Pretreatment of cells with workshop-typed clone 9A6 moAb completely blocked binding of KOR-SA3544 clone in all 9 leukemic specimens and in granulocytes (data not shown).

### Cytoplasmic presence of CD66c in ALL blasts

**Figure 3:**
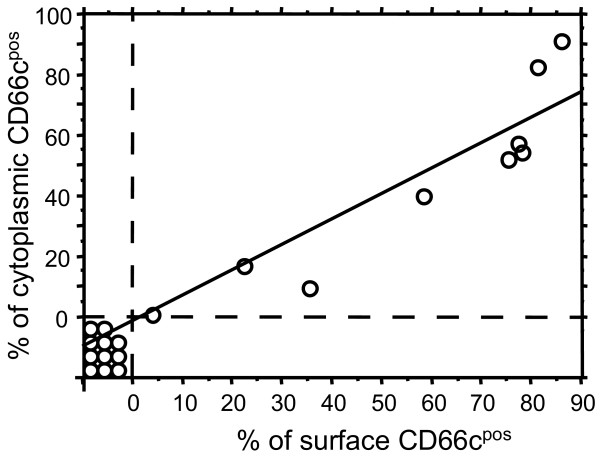
**Relationship of surface and cytoplasmic expression of CD66c. **Percentage of surface expression of CD66c in ALL blasts is plotted against cytoplasmic expression (after cell membrane permeabilization). Samples of 20 patients at ALL diagnosis are shown, 12 CD66c negative and 8 CD66c positive. Regression coefficient R^2 ^= 0.927

We have studied surface and cytoplasmic expression of CD66c in 20 ALL diagnostic samples by flow cytometry. In contrast to findings of Sugita et al [13], we have detected CD66c exclusively in all 8 surface positive cases. None of the 12 surface negative cases stained in cytoplasm (Figure 3). The probable cause of the opposite finding in several cases (lower percentage after permeabilization than on surface) is a higher background after permeabilization (isotypic control mean fluorescence intensity was 4.3 ± 2.0 and 9.7 ± 3.7 for surface and permeabilized staining, respectively), which covers borderline events.

### Transcription of CEACAM6 gene

**Figure 4:**
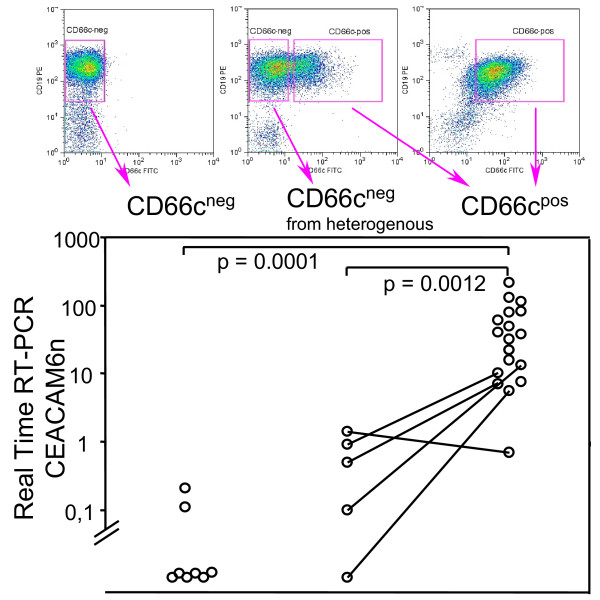
**Transcription of CEACAM6 versus surface CD66c expression on sorted cells. **FACSsorted CD66c surface negative (CD66c^neg^) or positive (CD66c^pos^) ALL lymphoblasts, five patients with heterogeneous CD66c expression were sorted into both CD66c negative and CD66c positive fraction (lines connect sorted fractions from the same specimen). Mann-Whitney test was used to compare groups (n = 32). CEACAM6n value is normalized to beta-2-microglobulin (see Methods).

To extend the above findings, we used Real-Time Quantitative Reverse Transcription-PCR (RQ-RT-PCR) to quantitatively assess presence of specific CEACAM6 mRNA. We FACS-sorted CD19^pos^CD66c^neg ^or CD19^pos^CD66c^pos ^blast cells for RQ-RT-PCR analysis. We didn't find significant amount of CEACAM6 transcript in surface CD66c^neg^lymphoblasts, whereas CD66c^pos ^cells contained CEACAM6. When CD66c^neg ^and positive fraction was FACS-sorted of heterogeneous specimens (lymphoblasts partly positive for CD66c) the level of CEACAM6 was observed higher in CD66c^neg ^cells and lower in CD66c^pos ^cells as compared to uniform populations (Figure 4). In one specimen (ALL patient with Down syndrome), CEACAM6 wasn't increased in CD66c^pos ^fraction.

### Western blot

**Figure 5:**
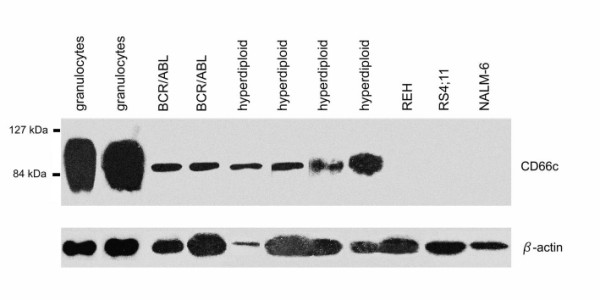
**Western blot **of granulocytes, ALL samples of CD66c positive cases and surface CD66c^neg ^cell lines with TEL/AML1pos (REH), MLL/AF4pos (RS4;11) translocation and with no fusion (NALM-6).

We further question the intracellular CD66c positivity in surface CD66c negative cell lines. We performed Western blot as described by Sugita et al. [13] on REH (TEL/AML1^pos^) and RS4;11 (MLL/AF4^pos^) cell lines and found no CD66c protein (Figure 5). Furthermore we found NALM-6 (surface CD66c^neg^, no translocation) cell line negative. Two BCR/ABL and four hyperdiploid (all surface CD66c^pos^) diagnostic samples used as positive controls were positive, with the similarly narrow band contrasting to broad band detected in granulocytes (Figure 5), suggesting different glycosylation in keeping with report by Sugita.

### Stability of surface expression from diagnosis to relapse

**Figure 6:**
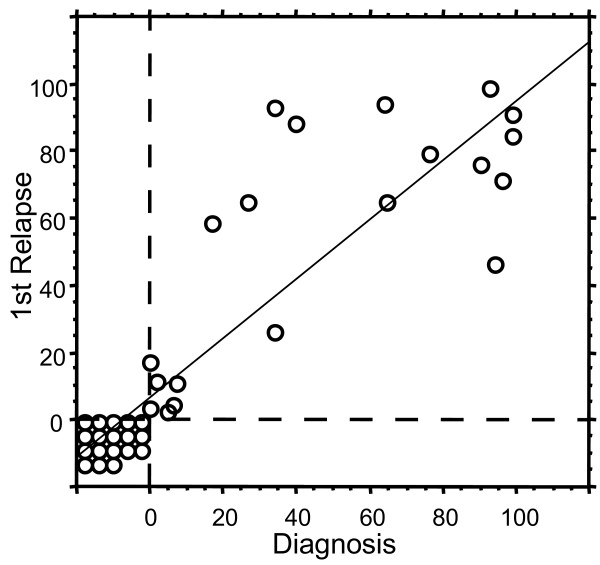
**Stability of CD66c from diagnosis to relapse. **Each circle represents one patient (n = 39). Percentage of CD66c^pos ^blasts at diagnosis is plotted against percentage of CD66c^pos ^blasts at relapse. Regression line with 95% confidence R^2 ^= 0.755

All relapsed patients up till 12/2003 with available information on CD66c expression at diagnosis and at relapse were used to assess stability of CD66c expression. Comparison of CD66c expression in 39 cases of relapsed childhood ALL cases to their immunophenotype at diagnosis revealed that both negativity and positivity of this antigen was retained from diagnosis to relapse (Figure 6; median time to relapse 2.5y min 0.3y, max 5.3y). Although the quantitative levels of CD66c expression differed in some patients (median difference 0.0%, standard deviation 21%), no case of CD66c complete loss or gain was found in our cohort.

### Prognostic significance of CD66c expression

**Figure 7:**
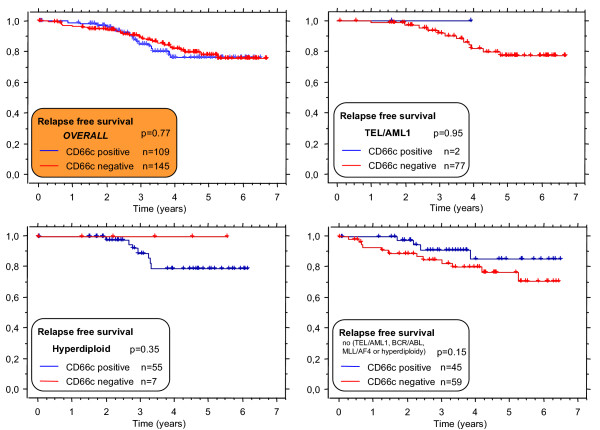
**Relapse free survival of cases with CD66c ^pos ^(blue line) or CD66c^neg^(red line) B-precursor ALL. **Unselected consecutive patients treated on ALL BFM95 protocol (median follow up 3.64 years). Since surface CD66c associates with genotype, separate analyses for distinct genotype subgroups are shown.

Only B-precursor ALL patients treated on the same ALL BFM 95 treatment protocol [20] (n = 254) were evaluated for prognostic impact. The prognosis did not differ for cases with either CD66c^pos ^blasts exceeding either 20% (Figure 7) or any other cutoff value tested (5%, 10% and 50%, data not shown).

Next, we asked whether CD66c expression correlated with the risk factors used in ALL BFM-95 protocol for stratification into risk groups [21]. No difference in relapse free survival (RFS) was noted when analyzed separately for each risk group or higher and lower initial leukocytosis (cutoff value: 2 × 10^4 ^cells per ml), age group or response to prednisone (groups as in Table 2).

When analyzed with respect to a genotype, we found no prognostic value of CD66c in any defined group (BCR/ABL^pos^, TEL/AML1^pos^, hyperdiploid ALL and none of the above-mentioned genetic changes, Figure 7 and Table 2). In contrast to the study by Hanenberg et al [22], there was no correlation between initial leukocytosis and CD66c in our cohort (Table 2).

## Discussion

Our data on childhood B-precursor ALL show that CD66c is more frequently expressed than the myeloid antigens included in the standard immunophenotyping panels for ALL. To our knowledge, CD66c is the most frequent myeloid marker in childhood ALL. This, together with the tight correlations between CD66c and genotype [5], makes CD66c a pertinent object of research on aberrant expression regulation.

In line with the data from Sugita, we confirm the specificity of KOR-SA3544 clone moAb for CD66c by CEACAM6 mRNA detection and by cross-blocking of KOR-SA3544 binding by representative 9A6 clone, that suggests a spatial proximity of the two epitopes recognized. Furthermore we show that all CD66c^pos ^ALL specimens show a similar extent of glycosylation as cell lines analyzed by Sugita, which differs from the extent of glycosylation in granulocytes.

Since there is a strong correlation of ALL genotype and CD66c expression, we hypothesized that surface CD66c expression would be controlled by gene transcription rather than by targeting to surface from intracellular stores as proposed by Sugita [13]. In accordance with this, both intracellular staining and Western blot failed to identify cytoplasmic CD66c protein in any surface CD66c^neg ^cells. Down the same line, no CEACAM6 transcript was detected in surface CD66c^neg ^lymphoblasts. Overall our data suggest that transcription is the checkpoint that leads to surface expression, rather then the former model, which proposed that all malignant lymphoblasts generate the CD66c molecule but only some of them target it for the cell membrane.

Interestingly, importance of this molecule was shown in a model of colorectal carcinoma where transfection with CEACAM6 inhibited anoikis (10), high CEACAM6 predicted high risk patients with resectable colorectal cancer (9) and CEACAM6 gene silencing decreased resistance to anoikis in vitro leading to inhibition of metastatic ability in mouse model (11). Although the function of CEACAM6 in ALL blasts is still unknown, this molecule's function has been recently associated with pathogenesis of other types of cancer in man [10-12,23,24]. Study of anti-CEACAM6 immunotoxin-based therapy in mouse model of pancreatic carcinoma was published recently [25].

So far, prognostic significance of expression of myeloid antigens CD13, CD14, CD33, CD65w, CD11b and CD15 has been studied with conflicting results (summarized in [26]). As determined in our large cohort of patients treated on ALL BFM 95 protocol, no prognostic significance of CD66c could be revealed in general, nor when we analyzed separate risk groups or TEL/AML1^pos^, BCR/ABL^pos^, hyperdiploid and other B-precursor ALL cases separately. Furthermore, instability of aberrant expression was reported for most myeloid markers (CD13, CD14, CD15, CD33 and CD65).

Stability of expression is a major concern of flow cytometric studies of MRD. In present, use of multiple CD markers is widely recommended to prevent MRD underestimation due to the immunophenotype shift (discussed in [15,27]). In current study we show for the first time that CD66c expression stays qualitatively stable from diagnosis to relapse in all relapsed cases studied. This finding, together with high frequency of CD66c^pos ^cases, supports inclusion of CD66c into a moAbs panels for MRD detection in patients positive for this CD marker at diagnosis. However, anecdotal downregulation of CD66c expression during chemotherapy has been observed [15], but has not been methodically studied yet. Any temporary downregulation might lead to falsely lower values of MRD measurement – thus, it would be worthwhile to disclose whether this phenomenon occurs regularly at certain points of chemotherapy.

Mutual exclusiveness of MyAg expression as well as different stability of CD66c compared to other MyAgs [28] challenges the general practice of prognostic evaluation of MyAg^pos ^ALL cases as a group [26] and favors individual evaluation of contribution/regulation of each MyAg for blast cell.

## Conclusion

CD66c presents some of the tightest associations with ALL genotype. Although our findings indicate that CD66c is unlikely to gain a practical importance as a prognosis predictor, there are several reasons to focus on it in diagnostic and MRD studies. CD66c, apparently the most frequently expressed aberrant antigen in childhood ALL, is very useful in discriminating leukemic blasts from non-malignant cells. Aberrant expression remains a puzzling phenomenon that warrants further investigation. If it is confirmed by techniques sensitive enough that the so called "aberrant markers" are truly not expressed on any subtle population of lymphoid precursors, there will be an opportunity to find new targets for specific ALL therapy (e.g. monoclonal antibodies against differently glycosylated form of CD66c) that will spare the non-leukemic precursors, thus reducing the treatment toxicity.

## Competing interests

The author(s) declare that they have no competing interests.

## Authors' contributions

TK performed flow cytometry, cell sorting, RQ-RT-PCR study and drafted the manuscript, MV carried out the Western blot study, EM acquired and analyzed patients flow cytometry data and performed the statistical analysis, JM designed and assisted to the RQ-RT-PCR study, JT designed RT-PCR, did the genotype detection and critically discussed the manuscript, JS contributed to the study design and organization and OH conceived of the study, analyzed data and drafted the manuscript. All authors read and approved the final manuscript.

## Pre-publication history

The pre-publication history for this paper can be accessed here:



## Figures and Tables

**Table 1:** Frequency of CD66c and myeloid antigen expression. Cases with >20% blasts are regarded positive, coexpression of CD66c and other MyAg is tested by Fisher's exact test.

Molecule	No of cases (total = 365)	Proportion [%]	Coexpression with CD66c	
CD66c	156	43		
CD33	85	23		
CD15	72	20		
CD13	57	16		
CD65	14	3.8		
CD66c and CD33	21	5.8	mutually exclusive	p = 0.002
CD66c and CD15	30	8.2	random	NS
CD66c and CD13	9	2.5	mutually exclusive	P < 0.0001
CD66c and CD65	2	0.55	mutually exclusive	p = 0.029

**Table 2:** Correlation between risk factors and CD66c expression. The distribution of CD66c^pos ^and CD66c^neg ^cases (cutoff 20%) is shown. In addition, no difference was observed in the RFS of the risk-defined subsets based on the CD66c expression (log-rank test p-value > 0.05 in all analyses). Only patients treated by a single ALL BFM-95 protocol are shown here (n = 254).

	CD66c^pos ^cases	CD66c^neg ^cases	p-value (chi-square)
All patients	109	145	N/A

Prednisone poor responder	9	12	n.s.
Prednisone good responder	100	133	

Initial leukocytosis = > 20 × 10^9^/L	28	44	n.s.
Initial leukocytosis < 20 × 10^9^/L	81	101	

TEL/AML1	2	77	P < 0.0001
BCR/ABL	7	1	
MLL/AF4	0	1	
Hyperdiploid	55	7	
Other genotype (not TEL/AML1, BCR/ABL, MLL/AF4 or hyperdiploidy)	45	59	

Age 1–5	59	88	n.s.
Age >5	50	57	

Standard risk group	40	58	n.s.
Intermediate risk group	54	72	
High risk group	15	15	
